# Monocular SLAM for Autonomous Robots with Enhanced Features Initialization

**DOI:** 10.3390/s140406317

**Published:** 2014-04-02

**Authors:** Edmundo Guerra, Rodrigo Munguia, Antoni Grau

**Affiliations:** 1 Automatic Control Department, Technical University of Catalonia UPC, 08028 Barcelona, Spain; E-Mail: edmundo.guerra@upc.edu; 2 Computer Science Department, CUCEI, Universidad de Guadalajara, 44430 Guadalajara, JAL, Mexico; E-Mail: rodrigo.munguia@upc.edu

**Keywords:** monocular SLAM, human-robot interaction, HRI, stereo matching, depth estimation

## Abstract

This work presents a variant approach to the monocular SLAM problem focused in exploiting the advantages of a human-robot interaction (HRI) framework. Based upon the delayed inverse-depth feature initialization SLAM (DI-D SLAM), a known monocular technique, several but crucial modifications are introduced taking advantage of data from a secondary monocular sensor, assuming that this second camera is worn by a human. The human explores an unknown environment with the robot, and when their fields of view coincide, the cameras are considered a pseudo-calibrated stereo rig to produce estimations for depth through parallax. These depth estimations are used to solve a related problem with DI-D monocular SLAM, namely, the requirement of a metric scale initialization through known artificial landmarks. The same process is used to improve the performance of the technique when introducing new landmarks into the map. The convenience of the approach taken to the stereo estimation, based on SURF features matching, is discussed. Experimental validation is provided through results from real data with results showing the improvements in terms of more features correctly initialized, with reduced uncertainty, thus reducing scale and orientation drift. Additional discussion in terms of how a real-time implementation could take advantage of this approach is provided.

## 1. Introduction

Sensors are widely used in several scientific and technical fields like robotics enabling the perception of the environment and its elements surrounding the robotic systems. This has led to the development of several sensor-based problems within the field, such as simultaneous localization and mapping (SLAM). The SLAM problem states how a mobile robotic device can operate in an *a priori* unknown environment by means of only onboard sensors to simultaneously build a map of its surroundings and use it to track its position. Thus, the SLAM is one of the most important problems to solve in robotics heavily related with sensors and its applications.

Many approaches have been developed to deal with the SLAM problem, based on a wide selection of sensors and combinations of them. Generally speaking, exteroceptive sensors can be used to solve both mapping and localization, while proprioceptive sensors are only able to deal with localization. This difference makes exteroceptive sensors more useful in the SLAM context, and if the mapping is a requirement to deal with in any given approach, it will forcibly include this kind of sensors. In [1,2] many types of available sensors are discussed and the main drawbacks are commented. These characteristics and the continuous development of better yet cheaper camera devices produced a surge in camera-based SLAM works during the last decade.

The camera, as a sensor, provides huge amounts of data and information, which can be used to deal with several problems using techniques and approaches developed in computer vision. The extraction of features and data association problems can be treated with relative ease in a camera-based SLAM. The main issue with the utilization of cameras for SLAM is the depth estimation. Since each pixel on a camera sensor maps the view from a ray, its depth information cannot be obtained directly. The only way to obtain depth estimations with visual information is through triangulation, relying on at least two different images. This can be achieved in two ways: images from a single camera separated spatially and temporally, thus, producing the monocular approach; or taking images with different cameras simultaneously, producing the stereo vision approach. The latter approach relies generally in the epipolar geometry [3], and normally implies creating a system where the different cameras (as it is not necessarily restricted to two cameras) are of similar characteristics and calibrated. Besides, these systems follow a set of restrictions to optimize the data association exploiting epipolar geometry features. Those characteristics make the stereo SLAM an easier problem, with the weaknesses of requiring more computational power to deal with two or more video sequences at the same time, and the limit to the maximum depth that can be estimated imposed by the baseline of the rig.

On the other hand, monocular SLAM approaches constitute more complex problems normally, especially if the six DoF have to be recovered from an only-bearing sensor. Most of the approaches originated in robotics have been produced through filtering techniques, many of them derived from the Extended Kalman Filter (EKF). Many notable works have used other sensors besides the camera to help to estimate the parallax in order to find the depth value [4,5]. In [6,7], a good survey of approaches to SLAM is presented where several methods with different filtering techniques are discussed. Still, some of the most relevant works on SLAM are based on EKF, like the feasibility of real-time EKF monocular SLAM [8], the development of the inverse-depth (I-D) feature parametrization model [9], and more recently real-time relocalization [10,11].

The analogous problem in computer vision, known as structure from motion (SfM), has produced several solutions over time [12]. Although originally they were conceived as off-line solutions given their reliance on global non-linear optimization, these solutions eventually led to the creation of the keyframe methods. Though these methods are gaining popularity, they still rely on bundle adjustment [13], and so they are not the best option when the computational power might be a problem [14], and as they estimate the map only using some of the frames [15], a filtering approach able to reject data association errors [14] can produce maps with the similar levels of accuracy at a lesser cost.

A growing field in robotics research deals with the interaction of human and robotic devices, known as human-robot interaction (HRI) [16]. These trends also affect SLAM, as several recent works reveal, like exploration of large areas with a wide group of people and robots [17], or mapping a building explored by a human [18]. While this implies increased complexity in several aspects, like large maps management or data fusion from very different sensors, it also opens new strategies and approaches to several SLAM problems. In a collaborative context, monocular SLAM could be improved in terms of depth estimation of long range features, which both in the delayed [1] and undelayed approaches [19] tend to be difficult, as well as the initialization of a metric scale, which normally requires the introduction of a known scale information, *etc*.

In this work, authors present a collaborative framework for local scale SLAM based on previous works. Thus, the approach presented and discussed in [1,2,20,21], the delayed inverse-depth monocular SLAM (DI-D SLAM), is enhanced by enabling the utilization of data obtained through a different monocular sensor. This monocular camera is assumed to be worn by a collaborating human, which helps the robotic camera performing SLAM to explore a particular unknown environment. Section 2 briefly describes how the DI-D monocular SLAM works using HOHCT validation, detailing opportunities to improve it. Section 3 presents a brief discussion about stereo estimation, considering the characteristics found in our approach (non-constant, approximately known but variable calibration), and justifying the approach taken, before discussing which issues have been solved and the way to do so. The first issue solved is the metric scale initialization, and the second improved characteristic is the problem appeared when initializing features within the direction of movement. Section 4 describes the built experimental setup and some of the experiments, along with the assumptions done during data capture. This is followed by comments and discussion on the obtained results: achieved improvement, theoretical costs associated to those improvements, and the possibilities of a real-time implementation, describing possible architectures and optimizations.

## 2. Monocular SLAM with DI-D Initialization

**Figure 1.**
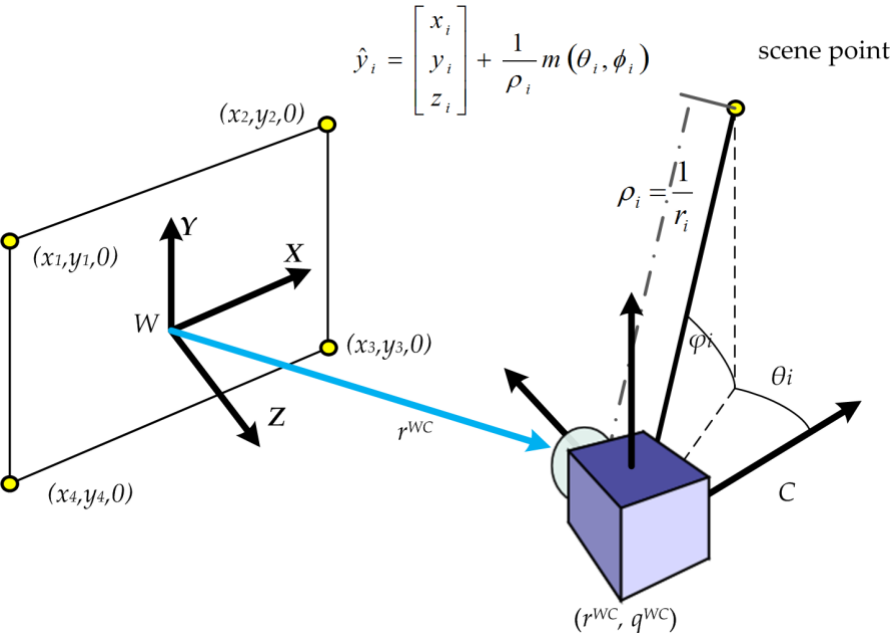
Inverse-depth problem with PnP scale initialization, adapted from reference [2].

The procedure for local monocular DI-D SLAM with a pinhole camera can be summarised in the terms of Algorithm 1. Based on the inverse-depth parametrization, it tracks landmarks through frames until they are initialized. The augmented state vector, **x̂**(Equation (1)), required by the EKF, is used to maintain the monocular camera position and the map. The first part of this column vector contains a vector **x̂_v_** that represents a robotic camera device, describing its pose and movement speeds (Equation (2)). The position of the camera optical centre is represented by **r***^WC^*, while its orientation with respect to the navigation frame is represented by a unit quaternion **q***^WC^*. Linear and angular velocities are described by ***v****^W^* and ***ω****^W^* respectively. The environment map estimation is represented by a set of *N* features **ŷ_i_** with i = [1, *N*]. Each feature **ŷ**_i_ is stored as a vector which models the estimated feature localization (Equation (3)) with respect to the world coordinates according to the inverse-depth model [9]. Coordinates *x_i_*, *y_i_*, *z_i_* are the optical center of the camera when the feature was seen for the first time; *θ_i_*, *ϕ_i_* represent azimuth and elevation for the ray which traces the feature point; depth *r_i_* to the feature is coded by its inverse ρ*_i_* = *1*/*r_i_*, see Figure 1.

(1) $$\hat{x}={\left[{\hat{x}}_{v},{\hat{y}}_{1},\ldots {\hat{y}}_{n}\right]}^{T}$$

(2) $${\hat{x}}_{v}={\left[\begin{array}{cccc} r^{WC} & q^{WC} & v^{W} & \omega^{W} \end{array}\right]}^{T}$$

(3) $${\hat{y}}_{i}={\left[\begin{array}{cccccc} x_{i} & y_{i} & z_{i} & \theta_{i} & \phi_{i} & \rho_{i} \end{array}\right]}^{T}$$

**Algorithm 1** Monocular SLAM with DI-D feature initialization (vid, refpose).
vid sequential image videoref_pose_ initial reference points
**begin** k: = 0  (x̂_0_, P_0_): = *Initialize* (*vid.FirstFrame*(), ref_pose_) **while** true **do**  img: = *vid.NextFrame*()  (x̂_k+1_, P_k+1_): = *StatePrediction*(x̂_k,_ P_k_)  (h_k_, ∇H_k_): = *MeasurementPrediction*(x̂_k+1_, P_k+1_)  (z_k_, S_k_): = *Matching&Validation* (h_i_, ∇H_i_, P_k+1_, img)  (x̂_k_, P_k_): = *Update* (x̂_k+1_ P_k+1_, h_k_, z_k_, S_k_)  (x̂_k_, P_k_): = *AddFeatures*(x̂_k_, P_k_, img)  k: = k + 1  **end while****end**


A metric scale initialization is required to estimate parallax in the DI-D monocular SLAM approach. Thus a set of accurate features are required initially, so that other new features can be initialized afterwards. This initialization process, based on the PnP problem [22], allows to set an initial value for the extrinsic camera parameters, and to map four points (see Figure 1). The problem of this approach is that those four points had to be coplanar, with known spatial relationships, and be able to be introduced manually on the algorithm or through artificially calibrated features guaranteeing to be seen at the start of the video sequence. Other common approach is producing unknown scale maps, introducing the scale later on. Both approaches are undesired for exploration tasks. Once the state is initialized, the EKF procedure is started. The camera is assumed to follow an unconstrained constant-acceleration camera motion prediction model [8,23], with Gaussian noise processes used to produce impulses with linear and angular speeds to move the camera, as seen on Equation (4):

(4) $$f_{v}=\left[\begin{array}{c} r_{k+1}^{WC}\\ q_{k+1}^{WC}\\ v_{k+1}^{W}\\ \omega_{k+1}^{W} \end{array}\right]=\left[\begin{array}{c} r_{k}^{WC}+(v_{k}^{W}+\upsilon_{k}^{W})\Delta t\\ q_{k+1}^{WC}\times q((\omega_{k}^{W}+\zeta_{k}^{W})\Delta t\\ v_{k}^{W}+\upsilon_{k}^{W}\\ \omega_{k}^{W}+\zeta_{k}^{W} \end{array}\right]$$

(5) $$P_{k+1}=\nabla F_{x}P_{k}\nabla {F_{x}}^{T}+\nabla F_{u}Q\nabla {F_{u}}^{T}$$

The features are assumed to remain static, which is the hypothesis generally used for mapping. As mapping dynamic features would damper the map, the validation of this used data association will remove them. The uncertainty of this prediction model is propagated through the covariance matrix (Equation (5)), where ∇*F_x_* and ∇*F_u_* are the Jacobian of the prediction model and process noise, respectively.

(6) $$h^{c}=\left[\begin{array}{c} h_{x}\\ h_{y}\\ h_{z} \end{array}\right]=R^{CW}\left(\left[\begin{array}{c} x_{i}\\ y_{i}\\ z_{i} \end{array}\right]+\frac{1}{\rho_{i}}m(\theta_{i},\phi_{i})-r^{WC}\right)$$

The observation model of a point **ŷ***_i_* computes a ray expressed in the camera frame as (Equation (6)), where **h**^C^ is observed by the camera through its projection in the image. *R^CW^* is the transformation matrix from the global reference frame to the camera reference frame. The Jacobian of the measurement model is computed at the same time, as it will be needed through the data matching phase. The matching process is performed through active search with a patch cross-correlation technique between images. The search is limited to elliptical search regions derived from the innovation matrix (Appendix, Equation (a4)). The set of pairing obtained are validated with a batch gating approach based on the “highest order hypothesis compatibility test” (HOHCT) [2,24], which makes an ordered search to validate the data association pairing using the Mahalanobis distance [25], outperforming the Joint Compatibility Branch & Bound (JCBB). The state and covariance update equations follow classical EKF formulation (see Appendix Equations (a1)–(a4)).

To add features to the state, the DI-D feature initialization is used, [1,26,27]. Based on stochastic triangulation, a hypothesis of initial depth for a feature using a delay is defined. To achieve this initialization a database of candidate feature points is created, and these landmarks are tracked until they achieve enough parallax to produce depth estimation, otherwise they are rejected. This parallax is estimated thanks to the initial metric scale initialization, which allows estimation of the camera trajectory. Still, this delayed approach can probe a weakness when the camera trajectory is not smooth enough to allow continuous tracking of features. Keyframe methods can implement techniques [28] to deal with these problems at the cost of higher computational requirements or even the introduction of additional sensors [29].

## 3. Feature Based Stereo Estimation of Depth

### 3.1. Stereo Discussion

Some of the weak points discussed before can be solved within a cooperative context exploiting data from another monocular camera. Assuming that the other camera device (the “free camera”, or C_f_) with known pose is near to the robotic camera performing SLAM (“SLAM camera”, or C_s_), joining observations from both cameras allow performing stereo-like estimation when their fields of view overlap. This way, a new non-constant stereo inverse-depth feature initialization approach will be used to address the issues.

Classical stereo approaches [30–32] rely on epipolar geometry to create a calibrated camera rig with multiple constraints. These constraints typically include that both cameras’ projection planes lie in the same plane in world coordinates, thus allowing optimization of the correspondence problem, as the match on an image of another's image pixel will lie in the corresponding epipolar line, and rectification can turn them into straight-lines parallel to the horizontal axis. Several works have dealt with rectification of stereo images for unrestricted pose cameras both calibrated [31] and uncalibrated [33,34].

**Figure 2.**
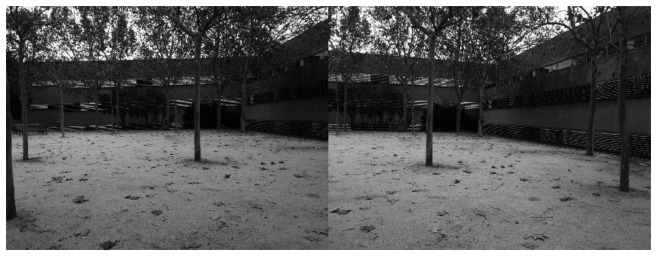
Pair of images captured at experimental environment.

**Figure 3.**
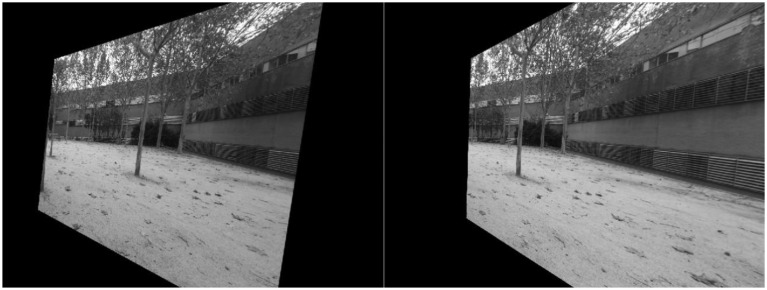
Pair of images rectified according to [31].

**Figure 4.**
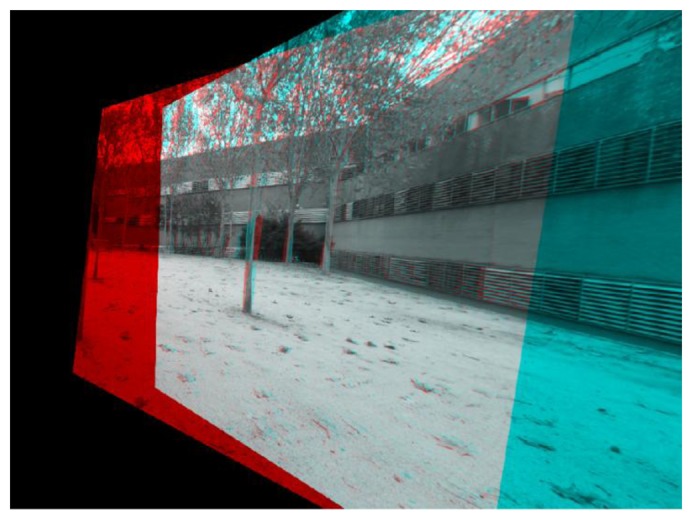
Stereo pair rectified and matched.

In [31], Fusiello detailed the first method to rectify stereo pairs with any given pairs of calibrated cameras. The method is based on rotating the cameras until they have one of their axis aligned to the baseline, and forcing them to have their projective planes contained within the same plane to achieve horizontal epipolar lines. Other works have proposed similar approaches to rectification of stereo pairs assuming calibrated, uncalibrated, or even multiple view [35–37] stereo rigs. These approaches need to warp both images according to the rectification found (see Figure 2 *versus* Figures 3 and 4), and in some cases producing great variations in terms of orientation and scale (Figure 4), thus rendering them less attractive in terms of our approach.

At any case, dealing with stereo features without rectified images is not a big problem in the proposed approach. As next subsections will describe further, the process of stereo features search and matching will be done sparsely, only to introduce new features: during the initialization, or when the filter needs new features. For both cases only a part of the image will be explored, and when adding new features in a system already initialized, additional data from the monocular phase can be used to simplify the process.

### 3.2. Initialization of Features with Non-Constant Stereo Estimation

**Figure 5.**
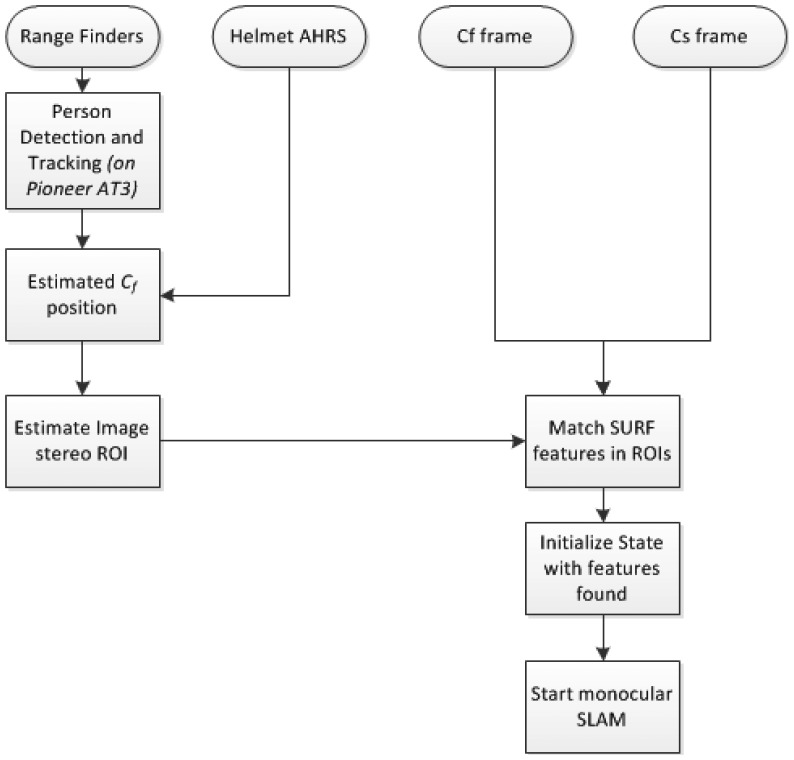
New metric scale initialization process.

**Figure 6.**
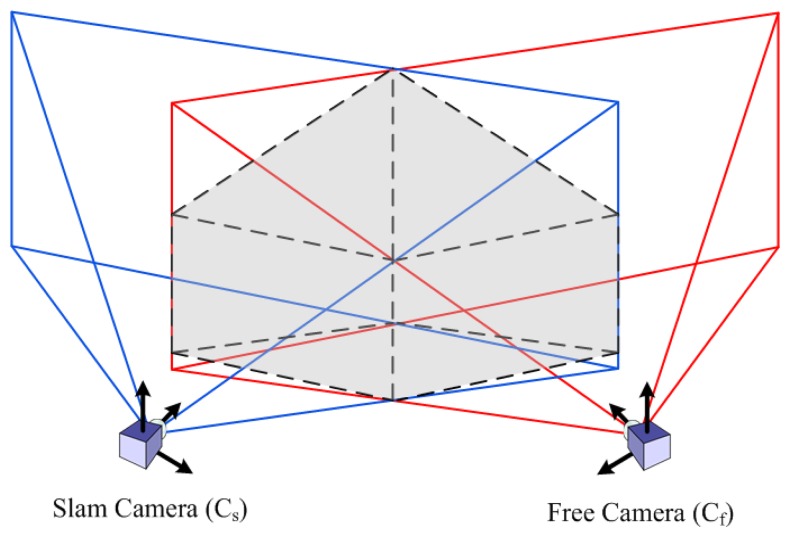
Polyhedron found intersecting fields of view until a depth where minimum parallax *pl_min_* could be found.

The requirement of metric scale initialization of the DI-D method can be easily solved under the cooperative assumption. Authors’ previous approach required the presence of a set of known, easily identifiable features to estimate them initially through the PnP problem. Then, assuming that at the start of the exploration a cooperating, free moving camera is near, the data from this camera can produce the features needed through stereo estimation. This process is shown in the diagram in Figure 5, where, after the poses of the SLAM camera C_s_ and the free camera C_f_ are known, the maximum distance from a camera where a point with a given minimum parallax (*pl_min_*) could lie is found. This distance is employed to build a model of the field of view of each camera, as a pair of pyramids, with each apex in the position of a camera, and the base centered along the view axis. Then it can be guaranteed that any point with parallax—between cameras—equal or greater than *pl_min_* will lie in the space intersected by the two fields of view modelled as pyramids, as seen in Figure 6. So the intersection between the different polygons composing the pyramids is computed as a set of segments (two point tuples), as described by Algorithm 2. Once all the segments are known, they are projected into the 2D projective space of each camera, and a search region is adjusted around them, determining the regions of interest where the stereo correspondence may be useful and significant.



**Algorithm 2** (ris, rif): = Find Stereo ROI (cams, camf, plmin).
cam_s_, cam_f_  SLAM camera, free camera (pose, intrinsic matrix)pl_min_     minimum parallax considered meaningfulris, rif    stereo ROI for images from cams and camf respectively
**begin** distance: = *FindDistance* (cam_s_.pose, cam_f_.pose) PyramidDepth: = *FindMaxDepth* (distance, pl_min_) Py1: = *ModelFoV*(cam_s_,PyramidDepth) Py2: = *ModelFoV*(cam_f_,PyramidDepth) intersection = Ø **for each** polygon_i **in** Py1  **for each** polygon_j **in** Py2   segment: = *Intersect*(polygon_i, polygon_j)   intersection.add(segment)  **end for****end for**ri_s_: = Ø; ri_f_: = Ø**if** (intersection = Ø) **then** ri_s_: = *Envelope*(*ProjectTo2D*(cam_s_.pose, intersection.points)) ri_f_: = *Envelope*(*ProjectTo2D*(cam_f_.pose, intersection.points)) **end if****end**


In the interest regions found, SURF-based feature descriptors [38] are matched to produce up to ten new stereo features to initialize the EKF state vector, see the end of the process at Figure 5. SURF is chosen over SIFT and FAST due to the more convenient trade-off offered in terms of matching accuracy and efficiency [39]. Each pair of matched features allows estimating the world coordinates of the feature point seen with simple triangulation, backtracking the point on the images from the SLAM camera and the free camera. Then, the set of landmarks found and estimated are introduced in the monocular EKF according to the inverse depth model.

### 3.3. Introducing New Features under Stereo

As described in [1,26], the DI-D initialization adds new landmarks into the map when a feature achieves parallax enough. This process may be easily disrupted if the features cannot be tracked long enough due to motion blur, illumination problems, trajectory irregularities, *etc*.,probably disrupting the filtering performance and convergence. The introduction of data association validation, though generally improves convergence of the filter [2,24], may also deprive the filter of features, as it may remove them faster that they are introduced. These problems can be mitigated under the assumption of a temporary stereo correspondence between cameras C_s_ and C_f_, introducing the features much earlier with accurate depth estimation, using a non-constant stereo I-D feature initialization approach.

**Figure 7.**
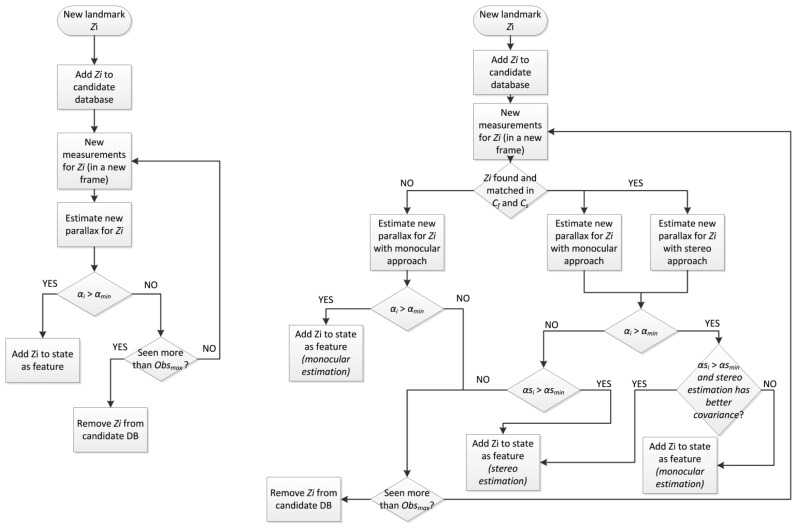
Feature initialization process according to the single monocular camera approach (**left**) and the proposed method with non-constant stereo I-D feature initialization (**right**).

Figure 7 shows the schemes for the single camera feature initialization process (Figure 7, left), and the new initialization process (Figure 7, right), with the help of the free camera C_f_. The approach shown on the left follows a classic strategy of storing and tracking candidate landmarks, detected through the Harris salience operator, to see if enough parallax is reached (α*_i_* > α*_min_*) within a given number of frames, and then to initialize them with an estimated depth value (with respect to the camera). On the proposed approach (Figure 7, right), if at prediction step stereo correspondence is found, the process to introduce new features will have different chances to introduce candidate landmarks as features. Those candidates that have enough parallax will be given preference to be inserted as full features (α*_i_* > α*_min_*). The depth estimation for these features will have the most available accuracy, obtained through parallax or through stereo estimation (available if α*s_i_* > α*s_min_*). Secondly, candidate landmarks which have not achieved enough parallax, but they are within the overlap region, are considered to be initialized as full features if they comply with depth estimation obtained through stereo matching. The last resource to initialize features would be finding new landmarks, and introduce them using only stereo matching, just like in the state initialization. These last two cases will also have to comply with restrictions (available if α*s_i_* > α*s_min_*) to guarantee a minimal accuracy when estimating the feature depth.

## 4. Experiments

The approach proposed in this work has been implemented in MATLAB^®^ to test and evaluate it. A set of five sequences corresponding to the same trajectory has been captured in a semi-structured environment. The sequences have been reduced to a resolution of 720 × 480 pixels and greyscale color, shortening the computational effort for image processing. Each sequence corresponds to a collaborative exploration of the environment at low speed, including a human and a robotic platform, each one equipped with the monocular sensors assumed earlier, C_f_ and C_s_, respectively. The data collected include monocular sequences, odometry from the robot, estimation of the human pose with respect to the robot, and the orientation of the camera for the five sequences.

**Figure 8.**
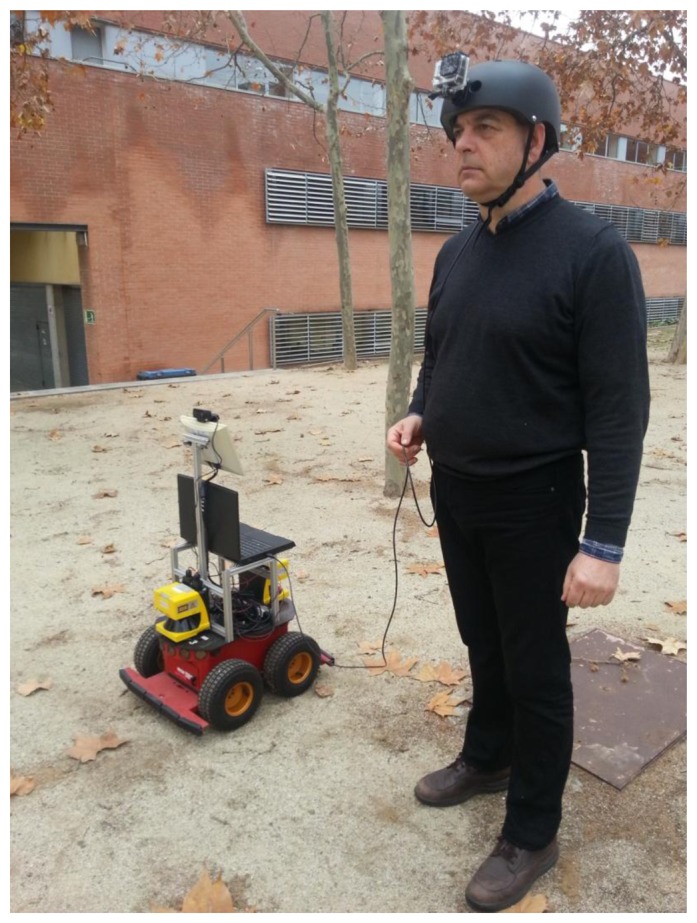
Robotic platform Pioneer AT3 with webcam and laser range finders. Helmet with camera and AHRS.

**Figure 9.**
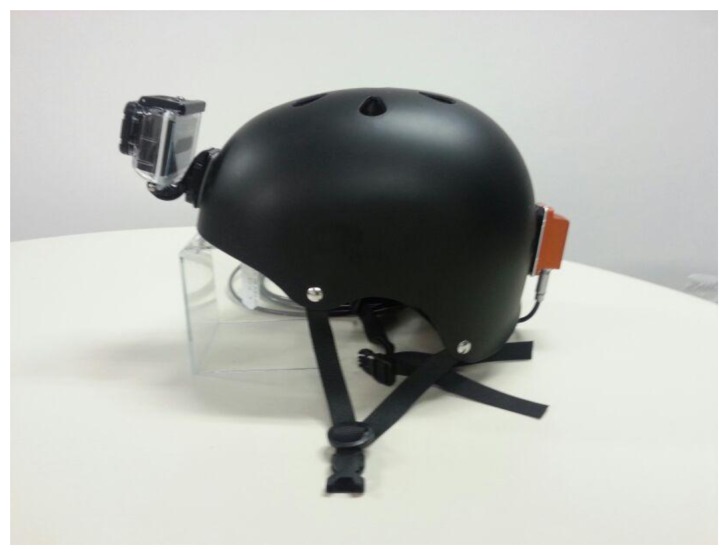
View of the helmet with the camera and AHRS unit placement detail.

The robot role is performed by a robotic platform based on the Pioneer 3 AT (Figure 8). The platform runs a ROS Fuerte distribution over an Ubuntu 12.04 OS. The platform is equipped with a pair of Leuzer RS4-4 laser range finders and a Logitech C920 webcam, able to work up to 30 frames per second (fps) at a resolution of 1,080 pixels. The sensors worn by the human are deployed on a helmet, including a GoPro Hero camera and an Xsens AHRS (Figure 9). All the data, except the GoPro camera video, have been captured and synchronized through ROS in the platform hardware. The ROS middleware provides the necessary tools to record and timestamp the data from the sensors connected to the platform. The C_f_ image sequence was offline synchronized with the C_s_ image sequence by marking frames with a laser pointer observed in both C_f_ and C_s_ sequences.

To estimate the pose of C_f_, orientation data from the AHRS are combined with the approximate pose of the human, estimated with the range finders [40,41]. The final position of the camera is computed geometrically as a translation from the estimated position of the atlas and axis vertebrae (which allow most of the freedom of movement of the head). These vertebrae are considered to be at a vertical axis over the person position estimated with the range finders, with height modeled individually for each person. In this work it is assumed that the environment is a flat terrain, easing the estimation process.

It is worth noting that for the proposed method, the pose of the camera worn by the human respect to the SLAM camera is not assumed to be perfectly known. Instead, it is considered that when needed, a “noisy” observation of the pose of C_f_ respect C_s_ is available by means of the methodology described above. The inherent error to the observation process is modeled assuming that the observation is corrupted by Gaussian noise. The value of the parameters used to model the inaccuracies for computing the pose of C_f_ were obtained statistically by comparing actual and estimated values. It is also important to note that an alternate method could be used for computing the relative pose of C_f_, for instance using different sensors. However, even with the use of a more reliable methodology the errors would not be completely eliminated.

**Figure 10.**
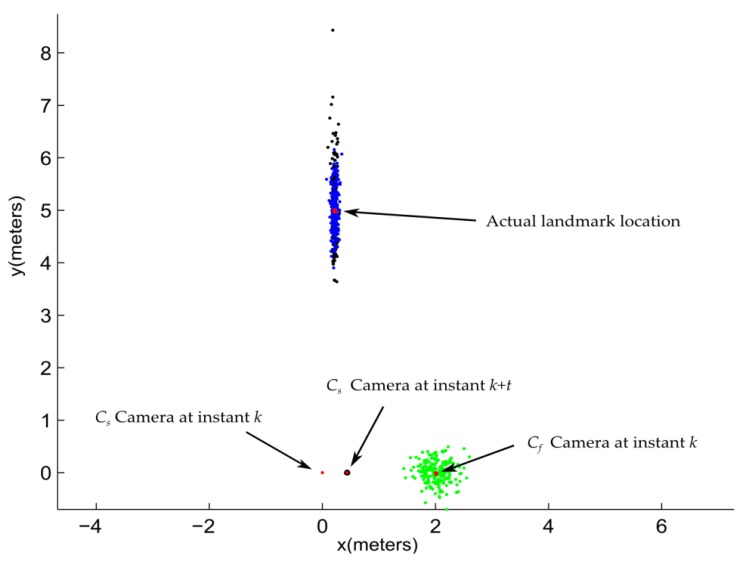
Initialization of a single landmark using: (i) the ID-delayed monocular method; and (ii) the pseudo-calibrated stereo rig approach. The actual position of the cameras and the landmark are indicated by red dots. Estimated locations of the landmark obtained by the ID-delayed method are indicated by the cloud of black points. Estimated locations of the landmark obtained with the pseudo-calibrated stereo rig are indicated by the cloud of blue points. Every green point indicates an observation of the C_f_ location.

On the bright side, the errors introduced by the pseudo-calibrated stereo approach are not as critical as one could be expecting. Figure 10 shows the results obtained from simulating the initialization of a single feature using: (i) the ID-delayed monocular method; and (ii) the pseudo-calibrated stereo rig approach. In the simulation, the C_s_ is located at [*x*, *y*] = [0, 0] at instant *k*. C_f_ is located at [*x*, *y*] = [2, 0] at instan *k*. Thus, it is assumed that the base-line between C_s_ and C_f_ is equal to 2 meters. A landmark is located at [*x*, *y*] = [0.21, 5]. For comparison purposes it is assumed that C_s_ was moved (at some instant *k* + *t*) to its right to [*x*, *y*] = [0.42, 0] in order to generate a parallax equal to 5 degrees. This amount is a typical value used as a threshold in the ID-Delayed method for initializing new features.

In the simulation, the drift associated with the estimated displacement of C_s_ is modeled by adding Gaussian noise with standard deviation σ = 1 cm to the actual location of C_s_ at instant *k* + *t*. The angular measurements provided by C_s_ are modeled by adding to its actual values Gaussian noise with σ = 0.5 degrees. In order to model the inaccuracies associated with the pseudo-calibrated stereo rig approach, the estimated location of C_f_ was modeled by adding a Gaussian noise with σ = 20 cm to its actual location. The errors introduced by the AHRS device have been taken into account by considering that the angular measurements provided by C_f_ are corrupted by Gaussian noise with σ = 1.5 degrees.

Using the above conditions, the location of the landmark has been estimated by triangulation with the location of C_s_ (at instant *k* + *t*) and with C_f_. In both cases the location of C_s_ (at instan *k*) was used as common pivot. The experiment was carried out 200 times.

For this experimental setup, even considering that the location of C_f_ is poorly estimated, the likelihood region obtained with the pseudo-calibrated stereo rig is always smaller than the likelihood region obtained with the ID-delayed approach. This is because landmark depth estimation is heavily dependent on parallax. In this case, for the pseudo-calibrated stereo rig, the parallax is about 22 degrees.

## 5. Results and Discussion

### 5.1. Experimental Results

The introduction of an auxiliary monocular sensor which can provide non-constant stereo information has proven itself useful. One of the weaknesses discussed on earlier works [2] was the need to manually introduce an initial metric scale, which has been removed. This grants more autonomy to the system, exploiting the implicit human-robot interaction without enforcing utilization of artificial landmarks. Besides, as the metric scale initialization can introduce more features into the initial state because is not limited to the artificial landmark, the scale propagates in a smoother way with reduced drift on the local scale.

**Figure 11.**
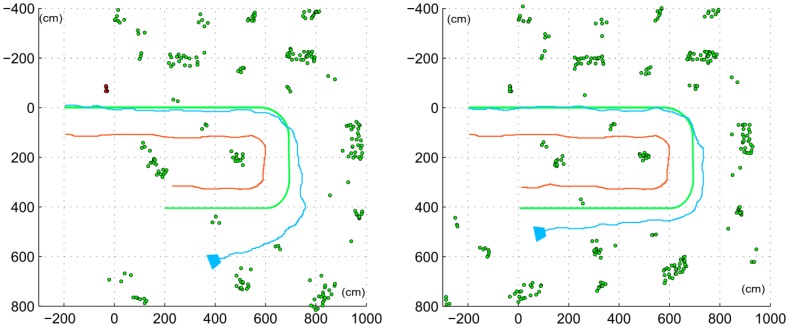
Trajectory estimated with classical DI-D monocular SLAM (**left**) and with the new non-constant stereo I-D approach. Green line denotes robot ground truth, orange line denotes C_f_ ground truth, and the estimated C_s_ trajectory is shown in blue. Red features (only in left plot) have been artificially calibrated and introduced to have an initial scale estimation in the DI-D approach.

**Figure 12.**
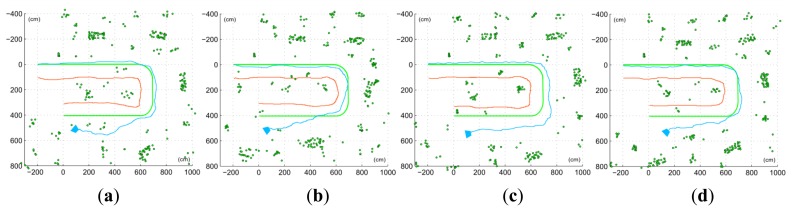
Estimations obtained for the rest of captured sequences, performing the trajectory several times and processed with the proposed approach.

Figure 11 shows results for one of the actual trajectories, with and without the utilization of the proposed non-constant stereo I-D feature initialization approach, right and left maps respectively. The introduction of stereo initialization of features allows introducing features with good depth estimation in a shorter time, thus making the system more resilient to quick view changes, such as turning. This can be seen on Figure 11 right, where the orientation drift is visibly minor. On the left trajectory estimation, the accumulated drift forces estimations so distant from actual observations within the data validation algorithm that most of the features are rejected. These rejections, combined with the drift itself, disrupt the estimation. On the other side, the trajectory estimated with non-constant stereo I-D feature initialization minimizes the drift and orientation deviation, thus keeping an accurate estimation even after the U-turn. Results obtained are consistent through all the 5 runs considered in the experimental setup, as shown on Figure 12 and Table 1.

**Figure 13.**
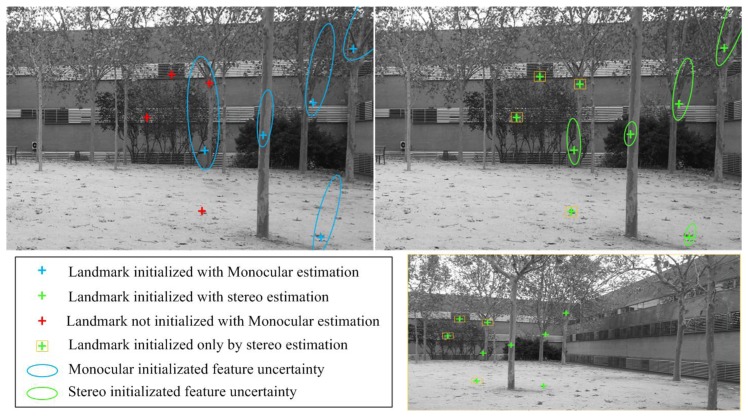
Landmarks initialized through stereo estimation. Left image: DI-D approach. Right top image: non-constant stereo I-D feature initialization. Right bottom image: image pair from C_f_ camera.

The introduction of the initialization of features with stereo estimation allows using more features that normally are rejected in the DI-D approach after being unable to achieve enough parallax. There are two cases were features are unable to achieve enough parallax: the first one is that they are distant features, and the camera would have to travel great distances in certain ways to see parallax; the second one affects features which lie in projective rays near the visual axis when camera moves forward. Several works [9,42] have dealt with distant features initializing them with heuristic values, as in an undelayed approach, as they tend to be useful to estimate orientation [26]. In the presented approach, the number of distant features used increases with respect to the DI-D approach, but those lying near the visual axis are the most benefit, as they will always present more parallax between C_f_ and C_s_. Figure 13 shows an example of a set of features initialized thanks to the stereo estimation. Note how several distant features could not be initialized under the DI-D SLAM, but were actually introduced under the non-constant stereo I-D approach. Besides, as it was detailed on Figure 7 (left) and commented above, the non-constant stereo I-D approach initializes features with the most accurate estimation.

## 5.2. Costs and Analysis

The apparent increase of the computational effort that would suppose the utilization of the presented approach could be hard to justify within the field of filtering based SLAM, which generally try to keep reduced computational costs. But the cost increase is limited and could be further reduced. For our C_s_ sequence set, made of a total of 7,120 frames in five sequences, only 38.22% (2,721 frames) presented a field of view overlap with the C_f_ camera. While this fact supposed an overhead of processing almost 40% more images, the exploration area was reduced with the search of the stereo ROI. With respect to this, it is interesting seeing how the newly proposed approach made less effort per feature to be initialized, compensating the bigger number of features used.

Table 2 shows the features used on each approach and the tracking effort required until the initialization of the features is done. Note how the non-constant stereo I-D feature initialization approach uses about 4% more features, but the time required to initialize them is smaller. This is because many features that are being tracked are instantly initialized through stereo once they lay in the overlapped field of view. This is advantageous because it allows to introduce features known to be strong (enough to be tracked) directly without more tracking effort, compensating the effort used for the C_f_ processing and stereo based initialization.

Furthermore, in real-time applications employing this technique, the C_f_ sensor could be upgraded to an “intelligent” sensor, with real-time processing capabilities. This approach would integrate image processing in the C_f_ sensor, allowing parallel processing of SURF features, and sending only extracted features, minimizing communications delay. This processing step could be done while the robotic camera C_s_ makes the general EKF-SLAM process, and thus it would be possible to have the SURF landmarks information after the EKF update, in time for the possible inclusion of new features.

## 6. Conclusions

A new approach for feature initialization in SLAM is presented, the non-constant stereo I-D feature initialization. This is based on the DI-D approach [1,2,20,21], and heavily focused towards human-robot interaction framework, under the form of a collaborative exploration of the environment. The human collaborates through a second monocular sensor with total freedom of movement and approximately known pose. As this monocular sensors moves freely, sometimes its field of view will be concurrent with the field of view of the camera doing monocular SLAM, producing non-constant stereo pairing. As the relative pose between the cameras and the calibration matrices of each one of them are known, the fundamental matrix of a stereo system can be found. Even though this allows using stereo rectification based on epipolar geometry, it proved inconvenient for our approach, and SURF-based feature matching is used when needed.

The introduction of the non-constant stereo has allowed improvements on the performance of two specific aspects in the local scale framework. Firstly, the introduction of an initial metric scale through synthetic features has been removed, substituted by the initialization of a set of features based on stereo estimation. This depth estimation has proven to have a slighted advantage: as the number of features introduced initially is not limited to four coplanar points, the use of a greater number of features placed at more varied distances makes the metric scale propagation smoother. Secondly, the introduction of later landmarks through stereo enables utilization of far distance features with real depth estimation, instead of the heuristically assigned value used in other works, and the initialization of frontal landmarks when the camera C_s_ moves forward. These changes have produced a locally strong and robust SLAM approach, thus enabling its future utilization on larger scale SLAM, as commented on [14]. This would further reduce the drift of the estimated trajectory, thanks to the covariance reduction of loop closure.

Once the viability of the proposed approach has been demonstrated, further research should focus on maximizing the advantages obtained from the HRI, while studying with more depth the impact to a system in terms of costs. In terms of exploiting the HRI, the stereo data, when available, should be used widely, evaluating the impact of its introduction during the measurement and update step of the EKF SLAM. This would probably require a full overhaul of the prediction and observation models currently used, but should improve significantly the accuracy of the approach. In line with this overhaul, the use of non-constant stereo enables to reinitialize a metric scale when the field of view overlaps, permitting the introduction of submapping techniques and other techniques to improve map management and achieving larger trajectories, including loop closing.

The proposed technique could be also expanded, with some modifications, to deal with more C_f_ agents, *i.e.*,a group of different humans could explore an environment accompanied by a robot mapping their surroundings with data from the sensors deployed on the humans. While this approach would require much more computational power and a more insightful architecture, it would be of great interest due its resemblance to hypothetical real cases, where not a human alone but a team, would explore new zones with robotic assistance. Besides, this approach could be evaluated and compared with robot network approaches, similar to [17].

## Figures and Tables

**Table 1.** Final pose estimation errors at the end of the trajectory.

**Experiment # and Figure**	**Classic DI-D Initialization**				**Proposed Initialization**			

	**|x| (m)**	**|y| (m)**	**d (m)**	**Angle (°)**	**|x| (m)**	**|y| (m)**	**d (m)**	**Angle (°)**
1 (Figure 11)	2.02	2.14	2.93	26	0.93	1.02	1.39	13.6
2 (Figure 12a)	1.53	2.92	3.29	−51	0.89	0.71	1.14	−29.4
3 (Figure 12b)	1.30	1.89	2.30	43	0.62	0.93	1.12	37.6
4 (Figure 12c)	3.41	3.17	4.64	77	1.07	1.38	1.75	14.2
5 (Figure 12d)	2.15	1.78	2.79	48	1.33	1.25	1.83	31.2

**Table 2.** Statistics of features used and frames being tracked until initialization.

	**DI-D Monocular SLAM**	**Proposed Initialization**
**Features initialized (total)**	1487	1549
**Features initialized (in average)**	297.4	309.8
**Average frames tracking a feature**	24.6	10.4

## Appendix: Nomenclature and Equations

### Nomenclature

Nomenclature used during the paper and annexes:

### Scalar

Scalar	
*x_i_*, *y_i_*, *z_i_*	camera coordinates
θ*_i_*, ϕ*_i_*	camera azimuth and elevation
*r_i_*	real depth to feature
ρ*_i_*	inverse depth to feature
α*_min_*	min. landmark parallax angle (monocular case)
α*S_min_*	min. landmark parallax angle (stereo case)
*pl_min_*	min. parallax to crop FoV intersection
Vector	
**x̂**	augmented state vector
**x̂***_v_*	robot camera state vector
**r***^WC^*	camera optical center position quaternion
**q***^WC^*	robot camera orientation quaternion
**V***^W^*	robot camera linear speed vector
**ω***^W^*	robot camera angular speed vector
*ŷ**_i_*	*i-th* feature vector
*f_v_*	camera motion prediction model
**υ***^W^*	linear speed pulse assumed by model
***ζ***^W^	angular speed pulse assumed by model
**h***^c^*	measurement prediction model
**g**	Kalman innovation vector
**z**	features measurement vector
**h**	predicted features vector
Matrix	
*Q*	process noise matrix
∇*F_u_*	process noise Jacobian matrix
*R^CW^*	world to camera transformation matrix
∇*H_k_*	measurement model Jacobian matrix
*S_k_*	innovation covariance matrix
*R_uv_*	measurement noise matrix
*W_k_*	Kalman gain matrix
*P_k_*	augmented state covariance matrix
∇*F_x_*	stated prediction model Jacobian matrix

### Equations

The following equations describe the update step of the Extended Kalman Filter used, mentioned in Section 2 (full details in [1]):

(a1) $${\hat{x}}_{k}={\hat{x}}_{k-1}+Wg$$

(a2) $$P_{k}=P_{k+1}-WS_{i}W^{T}$$

(a3) $$W=P_{k+1}\nabla H_{k}^{T}S_{k}^{-1}$$

(a4) $$S_{k}=\nabla H_{k}P_{k+1}\nabla H_{k}^{T}+R_{uv}$$
